# Fourier transform infrared spectroscopy for the distinction of MCF-7 cells treated with different concentrations of 5-fluorouracil

**DOI:** 10.1186/s12967-015-0468-2

**Published:** 2015-04-02

**Authors:** Bi-Bo Wu, Yi-Ping Gong, Xin-Hong Wu, Yuan-Yuan Chen, Fang-Fang Chen, Li-Ting Jin, Bo-Ran Cheng, Fen Hu, Bin Xiong

**Affiliations:** Department of Oncology, Zhongnan Hospital of Wuhan University, Hubei Key Laboratory of Tumour Biological Behaviors, Hubei Cancer Clinical Study Center, No. 169 Donghu Road, Wuhan, 430071 China; Department of Breast Surgery, Hubei Cancer Hospital, Wuhan, China

**Keywords:** FTIR spectroscopy, 5-fluorouracil, MCF-7 cell, Chemotherapy, Phosphatidylserine

## Abstract

**Background:**

In order to provide personalized treatment to patients with breast cancer, an accurate, reliable and cost-efficient analytical technique is needed for drug screening and evaluation of tumor response to chemotherapy.

**Methods:**

Attenuated total reflectance Fourier transform infrared spectroscopy (ATR-FTIR) was used as a tool to assess cancer cell response to chemotherapy. MCF-7 cells (human breast adenocarcinoma cell line) were treated with different concentrations of 5-fluorouracil (5-FU). The inhibition of cell proliferation was monitored by MTT, and apoptosis rates were determined by flow cytometry. Finally, spectra of the cell populations were acquired by ATR-FTIR.

**Results:**

The cell response to 5-FU was detectable at different concentrations by ATR-FTIR. First, a band observed at 1741 cm^−1^, representing membrane phospholipids, was enhanced with increasing 5-FU concentrations. In addition, the MCF-7 cell spectrum shifted progressively from 1153 to 1170 cm^−1^ with increasing drug doses. Finally, the normalized band intensity of 1741 cm^−1^/Amide I was highly correlated with the percentage of apoptotic cells as assessed by partial correlation analysis.

**Conclusions:**

These findings suggest that the effects of different concentrations of drugs can be monitored by ATR-FTIR, which may help evaluate the response to chemotherapy and improve treatment strategies.

## Background

Chemotherapy, together with surgery and radiotherapy, is the main treatment option for breast cancer. It is extremely challenging for clinicians to assess the chemotherapy effect, reduce side effects and design individualized regimens. At present, new drugs are evaluated for their potential to kill cancer cell lines, assessing cell viability by trypan blue exclusion [1], evaluating cancer cell growth inhibition by MTT (3-[4,5-dimethylthiazol-2yl] diphenyltetrazolium bromide) assay [2] or detecting the apoptosis rate by flow cytometry [3]. However, all these methods require laborious sample handling and staining. In addition, none of them provides a global view on the biochemical status of cells. The number of anti-cancer agents that fail in the clinic is by far more than that of products considered effective, suggesting that selection of drug molecules for clinical use needs optimization [4]. It is therefore urgent to develop accurate, reliable and cost-effective analytical techniques for pre-clinical screening of new candidate drugs and rapid evaluation of drug efficacy in patients with cancer [5].

Unlike traditional bioassays, Fourier-transform infrared (FTIR) spectroscopy is a vibrational technique that characterizes the chemical composition of a sample. FTIR spectroscopy has been widely used to analyze nucleic acids, proteins, phospholipids and other biological macromolecules. For example, the distinction of premalignant from malignant cells is possible using specific parameters obtained from Fourier transform infrared spectroscopy [6]. What’s more, this technique has been used to assess apoptosis in cancer cells after anticancer drug treatment [7], and distinguish drug-resistant from drug-sensitive cancer cells [8]. Moreover, FTIR spectroscopy of cancer cells in culture was proposed for monitoring and classification of anticancer drug effects [9]. Using FTIR spectrometry to monitor chemo-sensitivity of breast cancer MCF-7 cells treated with 5‐FU, decreased lipid utilization was observed with prolonged duration of drug action [10]. Interestingly, Hughes et al. could distinguish cell cycle signatures from cellular responses to chemotherapeutics by SR-FTIR spectroscopy [11]. El-Sherbeni et al. recently described the methanolic extract of stem bark as a strong cytotoxic agent against MCF-7 and VERO cell lines [12]. Attenuated Total Reflection (ATR) mode is another option for infrared spectroscopy, which allows a more precise localization of biochemical modifications and improves the distinction of the multidrug resistance phenotype [13]. It is not dependent on the thickness of the cell analyzed and only requires a small sample size, with easy sample preparation. In addition, it provides a more rapid, sensitive and simultaneous monitoring of different functional groups of macromolecules in biological systems [14]. The aim of this study was to explore the possibility of using ATR-FTIR to analyze and quantify the spectrum changes in MCF-7 cells exposed to different concentrations of 5-FU.

## Methods

### Cell culture and 5-fluorouracil treatment

MCF-7 cells were obtained from China Center for Type Culture Collection (CCTCC) and cultured in Dulbecco's Modification of Eagle's Medium (DMEM) supplemented with 10% Fetal Bovine Serum (FBS) and 1% penicillin-streptomycin solution at 37°C in a humidified atmosphere of 5% CO_2_. Phosphate Buffer Saline (PBS) was used for cell washing before subculture. Growth medium was changed every 48 hours and cells were detached by trypsinization using 0.05% trypsin-0.02% EDTA (Invitrogen). Cell culture medium, FBS and antibiotics were purchased from Hyclone (Logan, UT, USA). No live animals or human subjects were used in this study. 5-fluorouracil was obtained from Hubei Cancer Hospital in liquid form and diluted with 0.9% NaCl to 1000 μg/mL. This drug is routinely administered by infusion to patients with breast cancer.

### Cell viability assay

The overall cell growth was determined using the colorimetric MTT (3-[4, 5-dimethylthiazol-2yl] diphenyltetrazolium bromide) assay. Exponential phase cells were seeded in 96- well plates (5 × 10^3^ cells/well) and allowed to attach overnight at 37°C in a humidified atmosphere containing 5% CO_2_. Then, the cells were treated for 72 h with 5-fluorouracil at different concentrations, including 0, 0.78, 1.56, 3.125, 6.25, 12.5, 25, 50 and 100 μg/mL. The quantitation of viable cells was based on their capability to reduce the yellow MTT reagent to a purple formazan product, a reaction occurring in mitochondria. The cells were then incubated with 0.5 mg · mL^−1^ of MTT for 4 h at 37°C. The resulting formazan crystals were dissolved using formazan lysis liquid. The plates were left in cell incubator for 3–4 hours until formazan completely dissolved. After mixing on an orbital shaker, the number of living cells in treatment and control groups was directly proportional to the intensity of the purple product, which was quantitatively measured by a microplate reader (Enspire, PE) at 570 nm, according to the instructions provided with the kit. Each experiment was carried out in sextuplicate. The inhibitory rates were analyzed using ANOVA with the SPSS17.0 software. *P* < 0.05 was considered statistically significant.

### Apoptosis assessment by Annexin V-FITC/PI staining

Apoptosis is characterized by the translocation of phosphatidyl serine to the cell surface, and can be evaluated using an Annexin V-FITC antibody. Cells were seeded into 6-well plates (1 × 10^4^ cells/well), incubated overnight, treated with the indicated concentrations of 5-FU, and further incubated for 72 h. To assess apoptosis, cells were washed twice with ice-cold PBS (pH 7.4), resuspended in 1 × binding buffer, and incubated with Annexin V-FITC for 10 min in the dark. PI was then added for additional 5 min in the dark at room temperature. Annexin V-FITC and PI fluorescence were monitored using an FC 500 flow cytometry. Ten thousand events were collected per sample. Data were analyzed using the CXP analysis software.

### Sample preparation for ATR–FTIR spectroscopy

In a preliminary experiment, the degree of hydration strongly affected the FTIR spectral features of major cellular components. Therefore, cells were desiccated before analysis, according to the previously established method [15], which allowed reliable absorption signals in the region of 500–4000 cm^−1^. After overnight attachment, cells were treated with different concentrations of 5-FU for 72 h. Afterwards, the culture medium was collected and centrifuged, and cells were trypsinized for 1 min at 37°C. The reaction was stopped by adding 1 ml complete medium. Cells (4 × 10^6^) were collected by centrifugation (400 g, 5 min) and washed three times with sterile 0.9% NaCl solution to ensure complete removal of the growing medium. The resulting pellet was suspended in 5 μl of 0.9% NaCl in an Eppendorf tube and fixed with 70% ethanol for 30 min [16]. 70% ethanol was employed to maintain the stability of the cellular material although it is acknowledged may damage some component parts of biomolecular structures. Many different solvents can dissolve pure single lipid classes, but they are difficult in extracting complex lipids because strong forces of association cellular constituents, such as proteins and polysaccharides. What’s more, polar complex lipids do not dissolve easily in non-polar solvents. Lipid such as polyphosphoinositides are most likely bound to other cellular biopolymers by ionic bonds, and these are not easily disrupted by simple solvents [17]. Cells were dried under vacuum, and spread uniformly on the surface of diamond probe for ATR attachment (Thermo Scientific NICOLET iS10, USA): the probe was rotated to flatten the cells to form a film, which was analyzed without further manipulation. All spectra were recorded at a resolution of 4 cm^−1^ with 32 co-additions. Spectra were baseline corrected, normalized on the whole spectral window and the offset corrected.

## Results

### Effect of 5-fluorouracil on MCF-7 cell growth after 72 h of incubation

**Figure 1:**
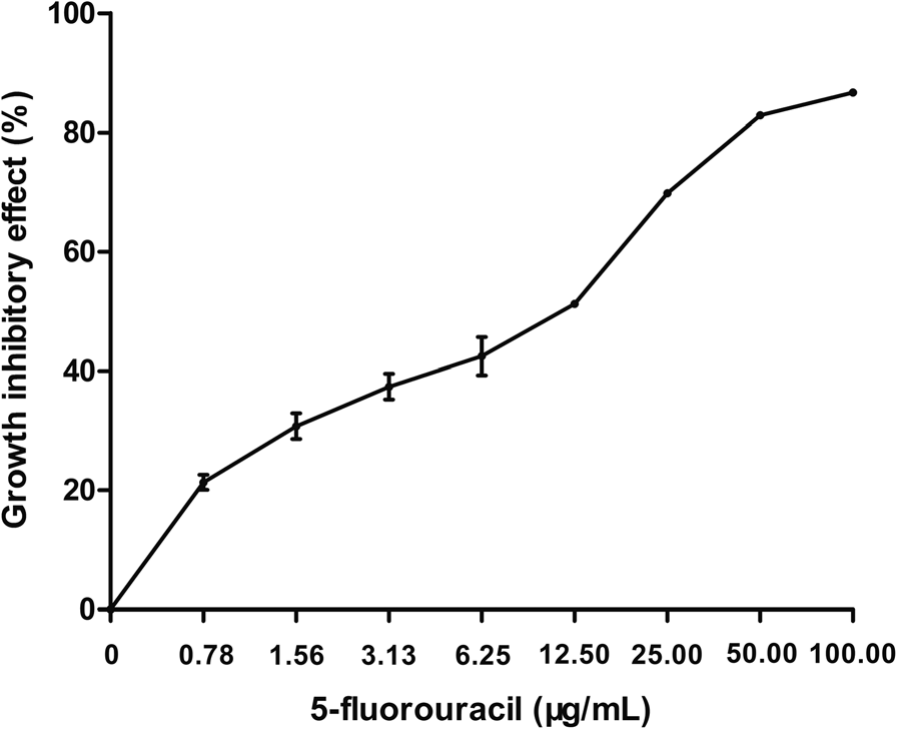
**5-fluorouracil mediates in vitro cytostatic activity.** Growth inhibitory effect of 5-fluorouracil on MCF-7 cells treated for 72 hours. Values represent the mean ± SEM of three independent experiments.

An MTT proliferation assay was carried out as previously described to assess the sensitivity of MCF-7 cells towards 5-FU. As shown in Figure 1, 5-FU overtly inhibited tumor cell proliferation, in a concentration dependent manner. The inhibition rates increased from 21.33 ± 1.25% to 82.84 ± 0.21% for drug concentrations between 0.78 and 50 μg/mL; no further increase was observed after treatment with 5-FU at 100 μg/ml compared with 50 μg/ml (86.69 ± 0.3% vs. 82.84 ± 0.21, *P* > 0.05).

### Apoptosis rates of MCF-7 cells after 5-fluorouracil treatment

**Figure 2:**
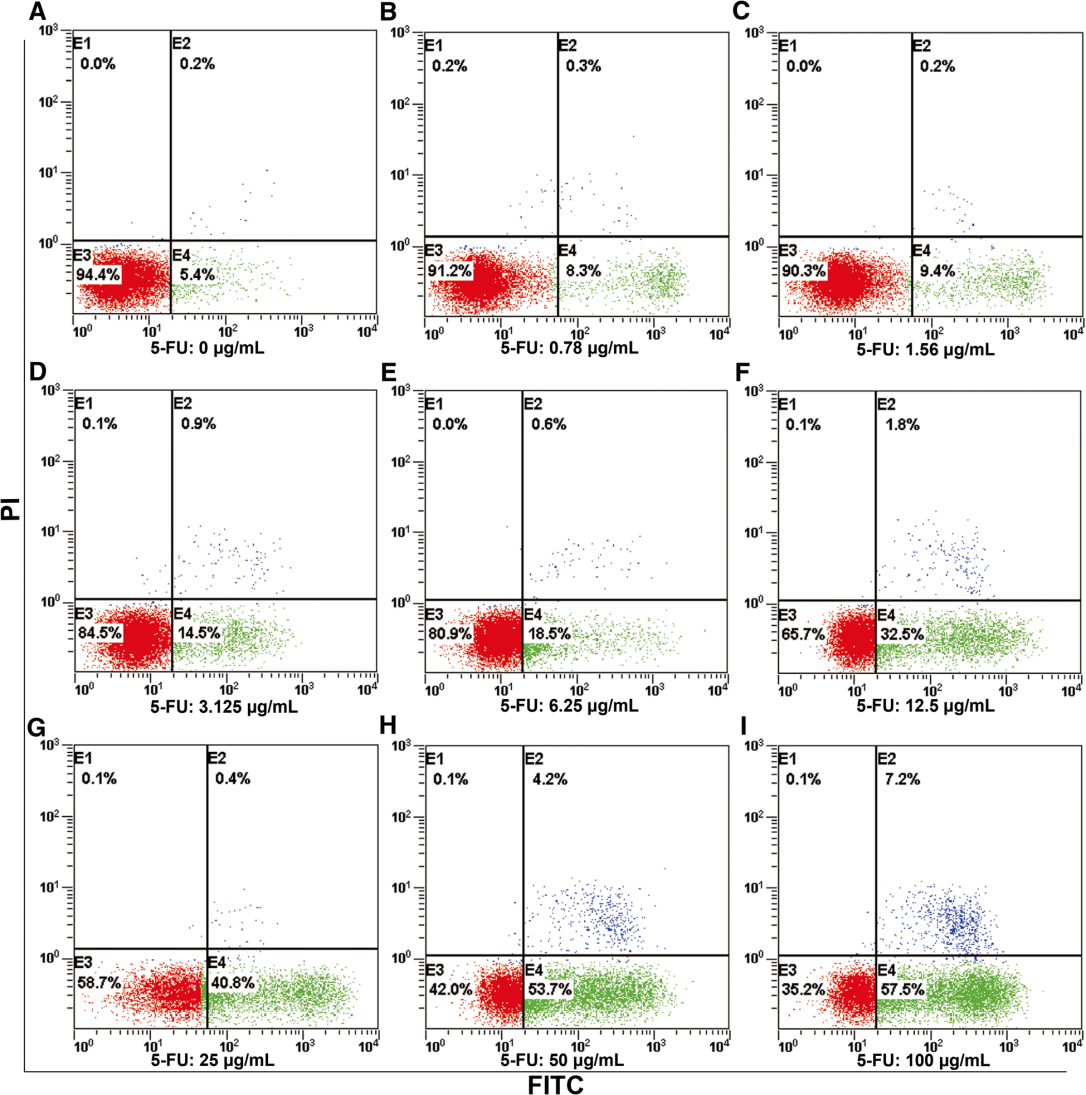
**Flow cytometry detection of Annexin V /PI-stained cells after treatment with different concentrations of 5-FU for 72 hours.** The sum of events in the upper right quadrant (late apoptotic/necrotic cells) and in the low right quadrant (early apoptotic cells) divided by the total number of events acquired. Panels **A** to **I** represent the results induced by different doses of 5-FU from 0, 0.78, 1.56, 3.125, 6.25, 12.5, 25, 50 to100 μg/mL separately.

We assessed the apoptosis rates by Flow Cytometry. In agreement with cell viability data, apoptosis rates were increased in 5-FU treated cells (Figure 2). PS is a negatively charged phospholipid normally predominant in membrane leaflets facing the cytosol. During cell apoptosis, the loss of membrane phospholipid asymmetry results in the exposure at the cell surface of phosphatidylserine, which can be labelled with Annexin V-FITC /PI. Untreated cells showed a baseline population of Annexin V-FITC positive cells at 5.23 ± 2.29%; however, the apoptotic index increased respectively to 32.3 ± 3.61, 42.4 ± 3.64, 56.27 ± 9.36, and 66.27 ± 1.38% after 72 h treatment with 5-FU at 12.5, 25, 50, and 100 μg/mL.

### Spectra of MCF7 cells treated with different 5-FU concentrations

**Figure 3:**
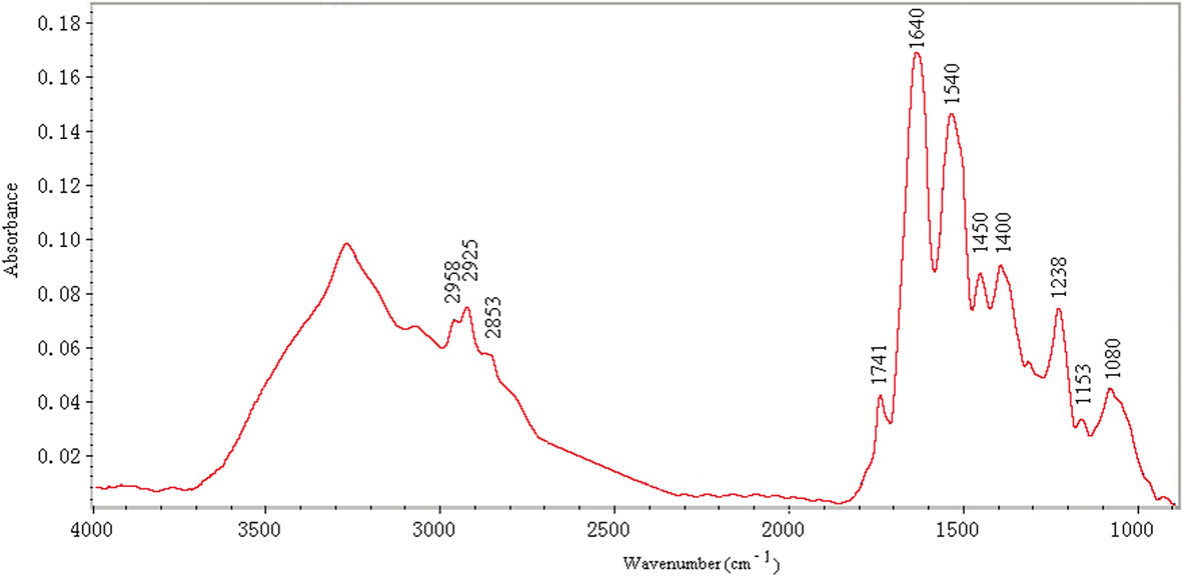
**Typical ATR-FTIR spectral signatures of MCF-7 cells treated with 5-fluorouracil.**

ATR-FTIR spectra of MCF-7 cells were obtained as described above. They were roughly similar in shape and contained two main regions (Figure 3). The absorptions in the 3200–3500 cm^−1^ region were not considered for further analysis due to the strong water signals. The signals at 2800–3000 cm^−1^ are dominated by symmetric and asymmetric stretching vibrations of CH2 and CH3 groups, mainly contained in fatty acids of cells [18].

The absorption signals between 1800 and 1700 cm^−1^ are characteristic of lipids as well. Indeed, the signal at 1741 cm^−1^ was attributed to an ester C = O stretching of phospholipids [19]. The region between 1700 and 1300 cm^−1^ is dominated by protein absorption; amide I stretching of carbonyl from peptide bond was observed at 1640 cm^−1^, a band sensitive to secondary structure of the protein. Deformation of protein amide N–H bond (Amide II) results in a signal at 1540 cm^−1^ [20]. The signals in the region between 1450 and 1400 cm^−1^ result from various amino acid side chains and some lipids (C–H bending vibrations of fatty acids at 1450 cm^−1^). Absorptions between 1300 and 900 cm^−1^ were mainly attributed to phosphate associated with nucleic acids, DNA and RNA. The absorption bands at 1238 cm^−1^ and 1080 cm^−1^ are characteristic of asymmetric and symmetric phosphodiester vibrations of nucleic acids [21]. The absorption signal at 1153 cm^−1^ was mainly attributed to the hydrogen and non-hydrogen bonds of the C—O stretching vibrations [22].

## Discussion

**Figure 4:**
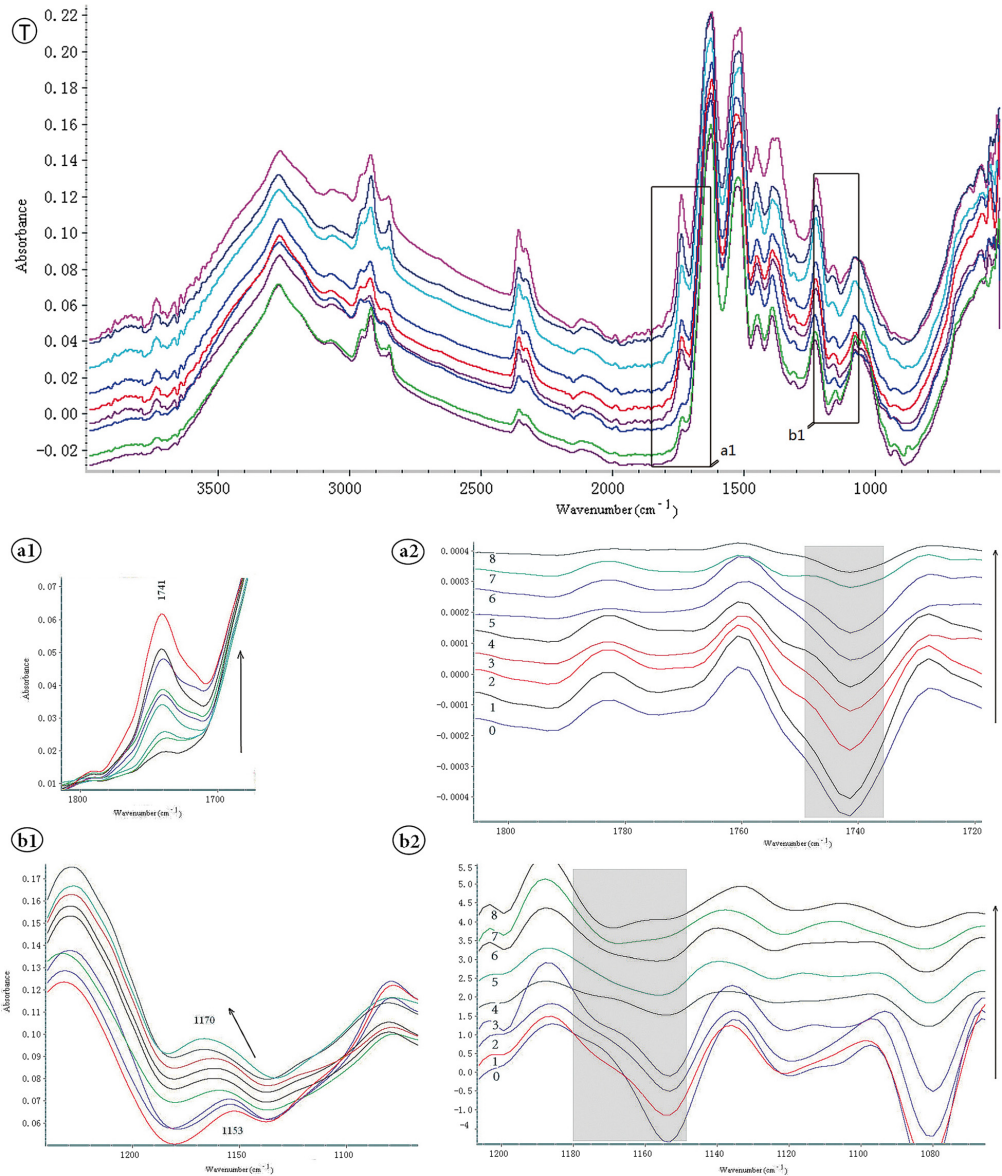
**ATR-FTIR spectra of MCF-7 cells treated with different concentrations of 5 -fluorouracil in Figure** 4 **T.** From bottom to top correspond to drug concentrations including 0, 0.78, 1.56, 3.125, 6.25, 12.5, 25, 50 and 100 μg/mL. The increase in the band at 1741 cm^−1^ for cells exposed to increasing concentrations of 5-FU shows in **a1** and the region at 1153–1170 cm^−1^ shows in **b1**. Corresponding secondary derivatives of the spectra are separately presented in **a2** and **b2** on the right.

In previous studies, the possibility to recognize cells with the help of infrared spectroscopy has been demonstrated. Hughes et al. were able to distinguish cell cycle signatures from cellular responses to chemotherapeutics by SR-FTIR spectroscopy [11]. Monitoring chemo-sensitivity of breast cancer cells by FTIR spectroscopy, they showed that MCF-7 cells decreased lipid utilization with prolonged drug action [10]. Here, we illustrated the distinction of the effects by different concentrations of 5-FU in MCF-7 cells. Of note, MCF-7 cells also showed decreased lipid utilization after treatment with 5-FU. There are two main discoveries in our study. One is that the signal at 1741 cm^−1^, representing membrane phospholipids, increased with the 5-FU dose (Figure 4a1). Since the peak at 1741 cm^−1^ was assigned to the ester C = O stretching of phospholipids and increased with dose addition, indicating that lipids utilization decreased during apoptosis. Secondary derivatives were also altered (Figure 4a2). Another critical finding concerns the new signal at 1170c m^−1^ in the spectrum of MCF-7 cells, which was increased with 5-FU concentration, shifting from 1153 cm^−1^ (Figure 4b1). Changes of secondary derivatives are shown in Figure 4b2.

### The 1741 cm^−1^ signal corresponds to phospholipid absorbance

The prominent band intensity changes were found around 1741 cm^−1^, which corresponds to the absorbance of phospholipids and lipids [23-25]. The signals at 3000–2800 cm^−1^ were associated with the acyl chains mainly present in lipids; interestingly, the discriminating power of the infrared spectroscopy appeared to become significant at angles less than 60 degrees, and even more significant when sampling deeper into cells, indicating that these lipids do not belong to the cell plasma membrane [13]. Plasma membrane lipids are mainly composed by phospholipids, which determine membrane stability, fluidity and enzymatic activity. The signal at 1741 cm^−1^ increased progressively with the 5-FU dose. Since the peak at 1741 cm^−1^ was assigned to the ester C = O stretching of phospholipids, these findings indicated increased lipid and esterified ingredients in the cell membrane as a result of 5-FU treatment. It also reflects the relative lipid content, indicating that lipids increase during apoptosis in these cells, in agreement with the morphological changes. MCF-7 cell volume was reduced after treatment with 5-FU, which resulted in a relative increase of the membrane surface area, causing a relative increase in lipid content [7]. However, it is not clear whether the intracellular membrane, or extracellular membrane, or both of them contributed to the spectra changes. These methods such as plasma membranes isolation, chromatographic analyses, and specific cell surface maker may help to clarify it.

### Changes of phospholipids may reflect cell viability

Multiple studies have reported increased lipid-related methylene absorbance after treatment with various apoptosis inducers, as observed in our experiments. At this time, the specific biochemical process responsible for the lipid-related FTIR spectral changes is unknown. Lipid changes, particularly phospholipid alterations in the cellular membrane, were shown to play a key role in the Bryo-1 induced differentiation and apoptosis of CLL cells. It is known that the contents of certain phospholipids in leukemia cell membrane are important for their viability [24]. The increased lipid content in two different leukemia cell lines (K562 and CEM) during apoptosis induced by VP-16 further highlights the role of lipids in the apoptosis process. In addition, membrane bound aggregated Aβ peptides might be formed in cellular membrane compartments, directly causing neuronal cell death [25]. MCF-7 cells stably transfected with miR-125b also showed decreased intensity of the band at 1744 cm^−1^, suggesting lower abundance of ester groups from triacylglycerols and cholesterol [26]. Pre-malignant and malignant cells were shown to have higher cholesterol requirements and therefore had high LDL receptor activity and increased cholesterol biosynthesis [27]. The lipid regions were shown to significantly increase in U937 cells treated with melphalan compared with untreated controls; meanwhile, treatment with the extracts of *C. formosum* and *P. kesiya* instead resulted in decreased lipids. The difference between melphalan-treated and control cells suggests an alteration in the lipid membrane associated with apoptosis, such as phosphatidylserine exposure [28]. Neuronal cells induced by C-terminal amyloid β-fragments also resulted in aggregation of the phospholipid membrane, with the band at 1737 cm^−1^ decreasing in intensity upon lipid/peptide ratio change [25].

### Changes of phospholipids may be associated with phosphatidylserine exposure

Apoptosis associated membrane changes, such as phosphatidylserine (PS) exposure and membrane blebbing could explain the increased C = O absorbance. PS is a negatively charged phospholipid normally predominant in membrane leaflets facing the cytosol. During cell apoptosis, the loss of membrane phospholipid asymmetry results in its exposure to the cell surface. As shown above, untreated cells contain a baseline population of Annexin V-FITC positive cells at 5.23 ± 2.29%; however, the apoptotic index increased to 32.3 ± 3.61, 42.4 ± 3.64, 56.27 ± 9.36, and 66.27 ± 1.38% after 72 h treatment with 5-FU at 12.5, 25, 50, and 100 μg/mL, respectively.

**Figure 5:**
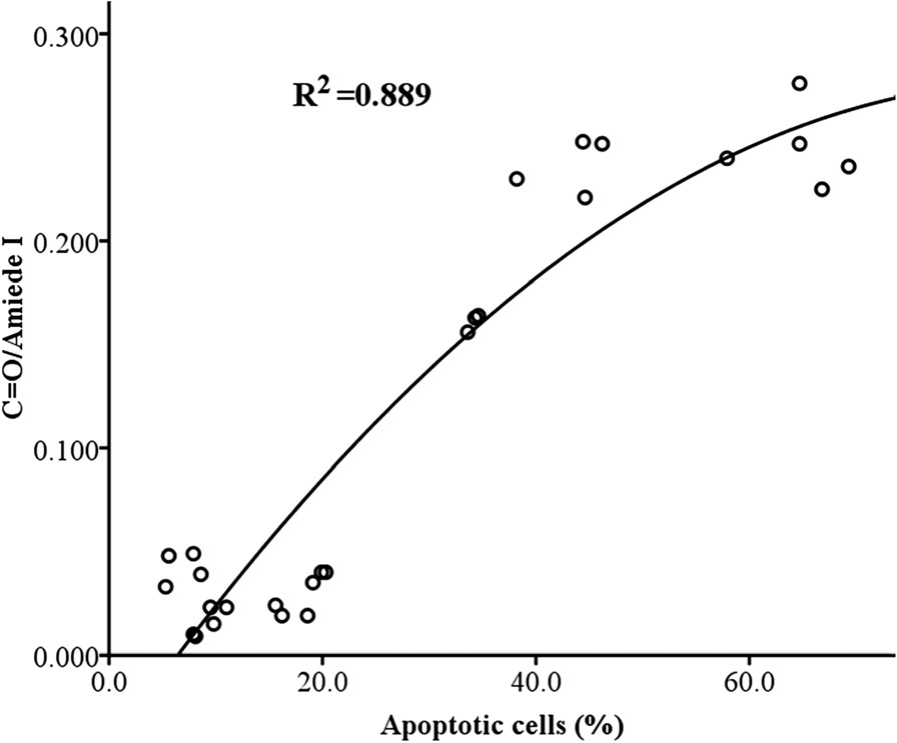
**Statistical correlation between the C = O/Amide I and apoptosis index (analysis performed from three independent experiments).**

The corresponding ATR-FTIR spectra showed a concentration dependent increase in the intensity of the band at 1741 cm^−1^ after 72 h treatment with 5-FU. The intensity of the latter band was correlated with apoptotic cell rate, as assessed by partial correlation analysis; the results from three independent experiments were plotted against the apoptotic cell rates obtained by Flow Cytometry. The C = O/Amide I ratio was correlated with the apoptotic index (R^2^ = 0.889) as shown in Figure 5. A plateau was observed with the 1741 cm^−1^/Amide I ratio measured for preparations containing 40% or 60% apoptotic cells. Similarly, a plateau was formed between 50 (82.84 ± 0.21%) and 100 (86.69 ± 0.3%) μg/ml of 5-FU treatment (72 hours) as detected by MTT. These findings indicate that these spectra may not only reflect cell apoptosis rate, but also includes information on cell growth inhibition. The comparison between data obtained with breast cancer cells, using combined measurements of ATR-FTIR spectroscopy and flow cytometry, confirms that apoptosis induces a conformational disorder of ester C = O stretching in phospholipids, as elsewhere reported, and allows the identification of a spectral marker of apoptosis that corresponds to phosphatidylserine externalization. Therefore, the contribution of C = O ester groups of phosphatidylserine to the absorption spectrum, added to that of other equally oriented phospholipids, increases the absorption intensity values of the signal at 1741 cm^-1^.

### A novel finding of progressive shift from 1153 to 1170 cm^−1^

Another significant finding is a new peak at 1170 cm^−1^ in the spectrum of MCF-7 cells which shifted progressively from 1153 cm^−1^ with increased concentrations of 5-FU. Absorption bands 1153–1170 cm^−1^ are mainly attributed to hydrogen and non- hydrogen bonds of C—O stretching vibrations [22]. These C—O groups mainly come from protein components, e.g. serine, threonine and tyrosine residues (C—OH); polysaccharide molecule C—O stretching vibrations are also included. These findings further indicated the important role of hydrogen bonding and glycosylation in MCF-7 cells during apoptosis. These three amino acids generally are substrates of phosphorylated protein kinase, suggesting that phosphorylation or dephosphorylation plays an important role in signal transduction in cells. The band at 1153 cm^−1^ in the spectra of cervical cells superimposed that of glycogen, whereas those at 1161 and 1172 cm^−1^ are mainly from the C-OH groups of serine, threonine, and tyrosine in proteins; the latter signals have been observed in colon tissues as well [29]. These authors indicated that the component bands at 1153 cm^−1^ and 1161 cm^−1^ are from the stretching vibrations of hydrogen-bonded C = O groups, whereas the band at 1172 cm^−1^ is due to the stretching vibrations of non-hydrogen-bonded C = O groups.

## Conclusions

In summary, the present study demonstrates that exposure of MCF-7 cells to 5-fluorouracil for 72 hours induces overall biochemical changes. We took advantage of the ATR–FTIR capability to pave the way for improved cell assessment after exposure to different concentrations of chemotherapy drugs. We propose that the drug induces alterations of phospholipid composition in the plasma membrane, which in turn increase the membrane permeability and fluidity. Further work is underway in our laboratory to clarify the metabolic processes behind these spectral changes. Nevertheless, our findings indicate that ATR-FTIR spectroscopy could be a useful tool for the assessment of tumor cell sensitivity to chemotherapy. Since this method provides distinctive biochemical information and requires only minimal sample handling and limited reagents, it constitutes a promising new alterative for monitoring cell alterations in the clinic.
